# Chemotherapy effect on myocardial fibrosis markers in patients with gynecologic cancer and low cardiovascular risk

**DOI:** 10.3389/fonc.2023.1173838

**Published:** 2023-08-08

**Authors:** Lu Ye, Dan-qing Wang, Meng-xi Yang, Qing-li Li, Hong Luo, Xiao-juan Lin, Ke-min Li, Liang Song, Yu Ma, Hui-qiong Huang, Lan Zhong, Lu Yang, Jian-jun Zhang, Feng-ming Gong, Hua-yan Xu, Lin-jun Xie, Ru-tie Yin, Ying-kun Guo

**Affiliations:** ^1^Department of Ultrasound, Key Laboratory of Birth Defects and Related Diseases of Women and Children of Ministry of Education, West China Second University Hospital, Sichuan University, Chengdu, China; ^2^Department of Gynecology, Key Laboratory of Birth Defects and Related Diseases of Women and Children of Ministry of Education, West China Second University Hospital, Sichuan University, Chengdu, China; ^3^Department of Radiology, Sichuan Cancer Hospital & Institute, Sichuan Cancer Center, School of Medicine, University of Electronic Science and Technology of China, Chengdu, China; ^4^Department of Radiology, Key Laboratory of Birth Defects and Related Diseases of Women and Children of Ministry of Education, West China Second University Hospital, Sichuan University, Chengdu, China

**Keywords:** cardiotoxicity, T1 mapping, myocardial fibrosis, cardiac magnetic resonance, neoplasms

## Abstract

**Background:**

Patients with gynecologic cancers experience side effects of chemotherapy cardiotoxicity. We aimed to quantify cardiac magnetic resonance (CMR) markers of myocardial fibrosis in patients with gynecologic cancer and low cardiovascular risk who undergo chemotherapy.

**Methods:**

This study is part of a registered clinical research. CMR T1 mapping was performed in patients with gynecologic cancer and low cardiovascular risk undergoing chemotherapy. The results were compared with those of age-matched healthy control subjects.

**Results:**

68 patients (median age = 50 years) and 30 control subjects were included. The median number of chemotherapy cycles of patients was 9.0 (interquartile range [IQR] 3.3–17.0). Extracellular volume fraction (ECV) (27.2% ± 2.7% vs. 24.5% ± 1.7%, *P* < 0.001) and global longitudinal strain (−16.2% ± 2.8% vs. −17.4% ± 2.0%, P = 0.040) were higher in patients compared with controls. Patients with higher chemotherapy cycles (>6 cycles) (n=41) had significantly lower intracellular mass indexed (ICMi) compared with both patients with lower chemotherapy cycles (≤6 cycles) (n=27) (median 27.44 g/m^2^ [IQR 24.03–31.15 g/m^2^] vs. median 34.30 g/m^2^ [IQR 29.93–39.79 g/m^2^]; P = 0.002) and the control group (median 27.44 g/m^2^ [IQR 24.03–31.15 g/m^2^] vs. median 32.79 g/m^2^ [IQR 27.74–35.76 g/m^2^]; P = 0.002). Patients with two or more chemotherapy regimens had significantly lower ICMi compared with both patients with one chemotherapy regimen (27.45 ± 5.16 g/m^2^ vs. 33.32 ± 6.42 g/m^2^; P < 0.001) and the control group (27.45 ± 5.16 g/m^2^ vs. 33.02 ± 5.52 g/m^2^; P < 0.001). The number of chemotherapy cycles was associated with an increase in the ECV (Standard regression coefficient [β] = 0.383, *P* = 0.014) and a decrease in the ICMi (β = -0.349, *P* = 0.009).

**Conclusion:**

Patients with gynecologic cancer and low cardiovascular risk who undergo chemotherapy have diffuse extracellular volume expansion, which is obvious with the increase of chemotherapy cycles. Myocyte loss may be part of the mechanism in patients with a higher chemotherapy load.

**Clinical trial registration:**

http://www.chictr.org.cn, identifier ChiCTR-DDD-17013450.

## 1 Introduction

Gynecologic cancers present a serious threat to the lives and health of women, and currently, the incidence of these cancers is increasing (1). Chemotherapy is one important treatment of gynecologic cancers (2). It is frequently complicated by the development of cardiotoxicity (3), which may lead to premature morbidity and mortality among cancer survivors (4, 5). Cardiotoxicity in patients with gynecologic cancer can be caused by some chemotherapy agents, such as taxane, platinum, anthracycline, cyclophosphamide, ifosfamide, fluorouracil, and bevacizumab (3). For the early detection of cardiotoxicity, noninvasive cardiac imaging should be considered (5). Cardiac magnetic resonance (CMR) is a multiparametric imaging modality that is changing clinical practice, and it has a wide variety of applications and enormous potential in cardio-oncology (6). CMR may be employed not only to evaluate cardiac structure and function but also to characterize myocardial tissue (7). Myocardial fibrosis due to collagen deposition from acute or chronic disease is associated with cancer treatment (8, 9). Using T1 mapping techniques, quantitative assessments of interstitial myocardial fibrosis may be performed and are noted by increased native T1 and extracellular volume fraction (ECV) measures (10, 11). Diffuse myocardial fibrosis caused by anthracyclines has been identified by CMR in cancer survivors and in those currently undergoing treatment (9, 12, 13). However, there is a lack of reports on native T1 and ECV in patients with gynecologic cancer. Thus, this study aimed to characterize native T1 and ECV as candidate markers of myocardial fibrosis in patients with gynecologic cancer and low cardiovascular risk who undergo chemotherapy in comparison with healthy female controls. It also aimed to determine whether these markers are associated with myocardial functional changes and chemotherapy course.

## 2 Methods

### 2.1 study design

This study is part of a registered clinical research (registration No. ChiCTR-DDD-17013450, http://www.chictr.org.cn). It was approved by the authors’ institutional research ethics board, and written informed consent was obtained from all patients and healthy control subjects. For this single-center cross-sectional cohort study, we screened patients with gynecologic cancer and low cardiovascular risk undergoing chemotherapy in the Division of Chemotherapy and Radiotherapy in the Department of Gynecology between September 2018 and April 2021. The patients were eligible for study enrollment if they fulfilled the following inclusion criteria: (1) diagnosed (initially diagnosed or recurrent) with gynecologic cancer, (2) undergoing chemotherapy, and (3) between the ages of 18 and 75 years. The exclusion criteria were (1) preexisting cardiovascular risk factors or disease, including coronary heart disease, cardiomyopathy, valvular heart disease, congenital heart disease, pericardial disease, diabetes, and uncontrolled hypertension; (2) history of cardiotoxic medication or chest radiation for other diseases; (3) contraindications to CMR; and (4) poor CMR T1 mapping quality. CMR was performed in intermission of chemotherapy or progression free interval. Simultaneously, age-matched female healthy volunteers were enrolled as healthy control subjects. Control subjects were excluded if they had preexisting cardiovascular risk factors or disease, had contraindications to CMR, or if the quality of the CMR was poor. We defined the time between the first dose of chemotherapy and CMR as the posttreatment time. Patients with chemotherapy cycles of >6 were referred to as the high cycle group (highC), and patients with chemotherapy cycles of ≤6 were referred to as the low cycle group (lowC).

### 2.2 CMR

All CMR examinations were conducted on a 3.0 T scanner (MAGNETOM Skyra, Siemens Healthineers, Erlangen, Germany) equipped with an 18-channel receiver coil. The CMR protocols included cine,T1-mapping and late gadolinium enhancement (LGE) imaging. To quantify the cardiac structure and function, 8 to 12 continuous sections were obtained from the mitral valve level to the left ventricular (LV) apex in the short-axis view using a balanced steady-state free precession pulse sequence. The vertical two-chamber long axis and horizontal four-chamber cine series were scanned using the same sequences used with the short-axis images. Matched T1-mapping imaging sequences were performed at three standard short-axis levels (basal, middle, and apical) in the left ventricle. Native T1 mapping was performed before contrast administration using a modified Look–Locker inversion recovery (MOLLI) sequence (TE= 1.11 ms; TR= 2.71 ms; fip angle= 35°; slice thickness= 6 mm;matrix= 139× 192 pixels; FOV= 280× 224.6 mm^2^) with motion correction (14). Post T1-mapping images were obtained 10 min after intravenous injection of gadobenate dimeglumine at a dose of 0.2 mL/kg using a MOLLI sequence. All images were obtained during breath-holding in end-expiration, and electrocardiographic gating was employed. We obtained hematocrit for ECV computation at the time of intravenous line insertion for the CMR. Myocardial intracellular volume fraction (ICV) was calculated as 1-ECV and LV intracellular mass indexed (ICMi) as the product of ICV and indexed LV mass (LVMi). For LGE imaging, a segmented phase-sensitive inversion recovery sequence with turbo FLASH readout at 17–19 minutes post contrast was performed. LGE images were obtained in the two-chamber, three-chamber and four-chamber planes, and a continuous stack of short-axis planes with full left ventricular coverage.

### 2.3 Image analysis

We analyzed all CMR images using commercially available software (Cvi42 5.11; Circle Cardiovascular Imaging Inc., Calgary, Canada). In addition, we calculated the LV function parameters, including LV ejection fraction (LVEF), LV end-diastolic volume indexed (LVEDVi), LV end-systolic volume indexed (LVESVi), and LVMi. LV myocardial strain analysis was conducted by applying the CMR feature tracking to the acquired LV four-chamber, two-chamber, and short-axis cines at the basal, mid, and apical ventricular levels. The endocardial border was manually set in end-systole and end-diastole. Global longitudinal strain (GLS), global circumferential strain (GCS), and global radial strain (GRS) were calculated in three dimensions. We calculated the native T1 and postcontrast T1 values by drawing endo- and epicardial borders on a series of three short-axis pre- and postcontrast MOLLI images. The partition coefficient lambda (λ) and ECV were computed as follows: ECV = λ(1−hematocrit); λ = (1/T1 myocardium postcontrast − 1/T1 myocardium-native)/(1/T1 blood postcontrast − 1/T1 blood-native). ECV maps and values were automatically obtained. Based on LGE images, obvious patch of the myocardium observed on any image was accepted as local myocardial fibrosis after elimination of artifacts.

### 2.4 Statistical analysis

Statistical analysis was conducted using IBM SPSS Statistics (SPSS version 25, IBM Corp., Armonk, USA) and GraphPad Prism 7 (GraphPad Software, La Jolla, CA, USA). After testing for normality, continuous variables were presented as means ± standard deviations if normally distributed and as medians and interquartile range (IQR) otherwise. Categorical variables were expressed as counts and percentages of the total. The patients were compared with the controls. Two-tailed *t* tests were employed to compare continuous variables when the data were normally distributed. For nonnormally distributed data, we employed the Mann–Whitney *U* test for comparisons. To compare the parameters of three groups, ordinary one-way ANOVA analysis was employed when data were normally distributed and the Kruskal–Wallis test when data did not conform to normality or homogeneity of variance. Bivariate correlation analysis was performed using Pearson’s or Spearman’s method, as appropriate. Pearson correlation was used for normally distributed data, whereas Spearman correlation was used otherwise. Subsequently, the variables were entered into a multivariate linear regression model to identify the factors independently associated with the ECV or ICMi. *P* values < 0.05 were considered statistically significant.

## 3 Results

### 3.1 Baseline characteristics

**Figure 1:**
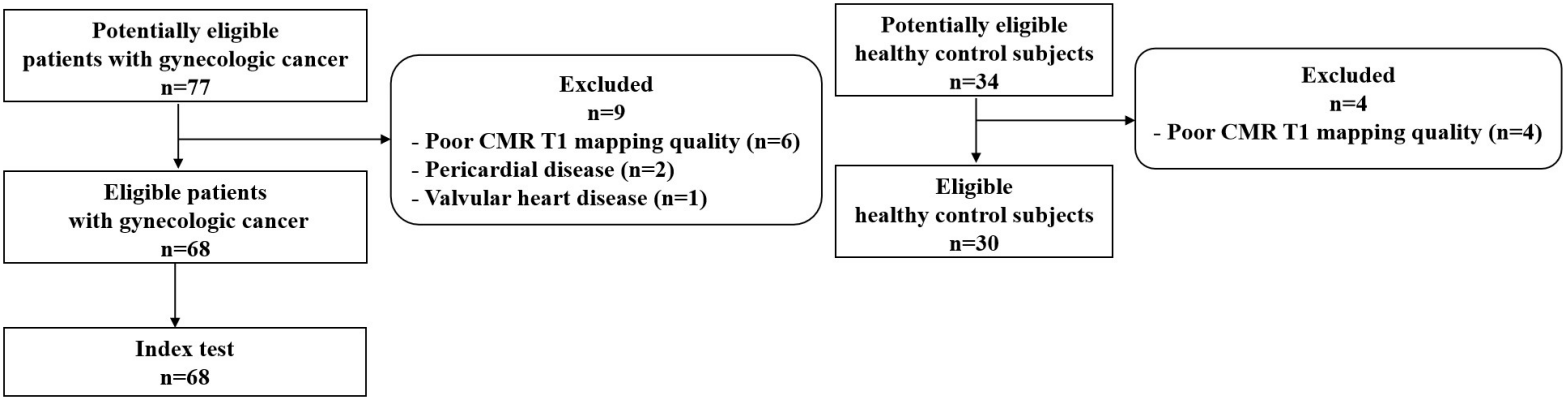
Study flowchat.

A total of 68 patients with gynecologic cancer and low cardiovascular risk as well as 30 age-matched healthy control subjects were included in this study (**Figure 1**). **Table 1** presents the general characteristics of both groups. The patients and the control groups did not differ in age, body surface area, or body mass index. The median age of the patients was 50 years (IQR 45–55 years). Cancer diagnoses included ovarian cancer (n = 36, 52.9%), fallopian tube cancer (n = 13, 19.1%), uterine or cervical cancer (n = 14, 20.6%), and trophoblastic tumor (n = 5, 7.4%). Among the 68 patients with gynecologic cancer, 55 patients (80.9%) received non-anthracycline chemotherapy; 13 patients (19.1%) received anthracycline-containing chemotherapy, all of which with low cumulative dose (<300 mg/m^2^ doxorubicin or <540 mg/m^2^ epirubicin). 38 patients (55.9%) received only one chemotherapy regimen, among which the taxane plus platinum regimen was the most commonly used (n = 31, 45.6%); 30 patients (44.1%) received two or more regimens. CMR was obtained at a median posttreatment time of 15.5 months (IQR 2.3–32.0 months). The median number of chemotherapy cycles was 9.0 (IQR 3.3–17.0). There were 27 patients in the lowC group and 41 patients in the highC group. The median number of chemotherapy cycles was 3.0 (IQR 1.0-4.0) in the lowC group and 15.0 (IQR 10.5-21.0) in the highC group.The median number of chemotherapy regimens was 1.0 (IQR 1.0–2.0). The number of chemotherapy regimens was found to be positively correlated with the number of chemotherapy cycles (*r* = 0.693, *P* < 0.001). The median interval from the last chemotherapy cycle and the CMR execution was 19.0 days (IQR 10.3-25.8 days).

**Table 1:** General characteristics of patients with gynecologic cancer and low cardiovascular risk compared with healthy control subjects.

	Healthy Control Subjects (n = 30)	Patients with Gynecologic Cancer (n = 68)	*P* value
Demographics			
Age at CMR (years)	42.5 (36.0, 54.8)	50.0 (45.0, 55.0)	0.136
Heart rate (beats/min)	70.5 (65.8, 73.6)	76.0 (69.4, 89.6)	0.007*
Body surface area (m^2^)	1.51 ± 0.13	1.54 ± 0.15	0.282
Body mass index (kg/m^2^)	22.61 ± 3.20	23.10 ± 3.75	0.536
Systolic blood pressure (mm Hg)	119 ± 9	118 ± 10	0.519
Diastolic blood pressure (mm Hg)	73 (65, 81)	79 (72, 85)	0.012*
CVD risk factors (n, %)			
Hypertension	0 (0%)	2 (2.9%)	0.999
Diabetes	0 (0%)	0 (0%)	0.999
Hyperlipidemia	0 (0%)	0 (0%)	0.999
Smoke	0 (0%)	1 (1.5%)	0.999
Medication (n, %)			
Calcium antagonist	0 (0%)	2 (2.9%)	0.999
ARB	0 (0%)	1 (1.5%)	0.999
Diuretics	0 (0%)	1 (1.5%)	0.999
Cancer diagnosis (n, %)			
Ovarian cancer	–	36 (52.9%)	
Fallopian tube cancer	–	13 (19.1%)	
Uterine or cervical cancer	–	14 (20.6%)	
Trophoblastic tumor	–	5 (7.4%)	
Cancer onset (n, %)			
Initial diagnosed	–	36 (52.9%)	
Recurrence	–	32 (47.1%)	
Number of chemotherapy regimens	-	1.0 (1.0, 2.0)	
Number of drug types	-	3.0 (2.0, 4.8)	
Posttreatment time (months)	-	15.5 (2.3, 32.0)	
Number of chemotherapy cycles	-	9.0 (3.3, 17.0)	
Chemotherapy drug type (n, %)			
Taxol + Platinum	–	56 (82.4%)	
Anthracycline	–	13 (19.1%)	
Cyclophosphamide/ ifosfamide	–	13 (19.1%)	
Bevacizumab	–	16 (23.5%)	
Others	–	6 (8.8%)	

Values are the mean ± SD, n (%), or median (interquartile range). CMR, cardiovascular magnetic resonance; CVD, cardiovascular disease; ARB, angiotensin receptor blocker.

* P < 0.05. - : no available data.

### 3.2 Ventricular volumes and function

**Table 2** present the difference in the CMR characteristics between healthy control subjects and patients with gynecologic cancer. LVEF was similar between the patients and control subjects (64.2% ± 7.7% vs. 63.7% ± 5.0%, *P* = 0.753). Four (5.9%) patients had an LVEF < 55%. LVEDVi, LVESVi, LVSVi and LVMi did not differ between the control subjects and patients. Patients with two or more chemotherapy regimens (n=30) had lower LVSVi compared with both patients with only one regimen (n=38) (36.2 ± 7.0 mL/m^2^ vs. 40.0 ± 8.2 mL/m^2^; *P* = 0.049) and the control group (36.2 ± 7.0 mL/m^2^ vs. 40.9 ± 4.0 mL/m^2^; *P* = 0.003).

**Table 2:** Cardiovascular magnetic resonance data of patients with gynecologic cancer and healthy control subjects.

	Healthy Control Subjects (n = 30)	Patients with Gynecologic Cancer (n = 68)	P value
Left ventricular function parameters			
LVEDVi (mL/m^2^)	64.4 ± 7.2	60.9 ± 16.1	0.259
LVESVi (mL/m^2^)	23.6 ± 5.2	22.5 ± 13.4	0.693
LVSVi (mL/m^2^)	40.9 ± 4.0	38.3 ± 7.9	0.104
LVMi (g/m^2^)	43.5 (36.7, 47.8)	41.5 (35.3, 47.2)	0.386
LVEF (%)	63.7 ± 5.0	64.2 ± 7.7	0.753
Left ventricular strain parameters			
GLS	−17.4 ± 2.0	−16.2 ± 2.8	0.040 *
GCS	−24.3 (−25.6, −21.8)	−23.4 (−24.8, −21.3)	0.068
GRS	43.3 ± 7.7	39.5 ± 8.9	0.054
Left ventricular tissue characterization			
LV Native T1 (ms)	1267 (1243, 1283)	1273 (1251, 1298)	0.176
LV ECV (%) ^a^	24.5 ± 1.7	27.2 ± 2.7	<0.001 *
LV ICMi (g/m^2^) ^a^	32.79 (27.74, 35.76)	30.46 (25.50, 36.40)	0.098

Values are the mean ± SD or median (interquartile range). ^a^Left ventricular tissue characterization data were available for 30 of 30 healthy control subjects and 58 of 68 patients with gynecologic cancer. GCS, global circumferential strain; GLS, global longitudinal strain; GRS, global radial strain; LV ECV, left ventricular extracellular volume fraction; LVEDVi, left ventricular end-diastolic volume indexed; LVEF, left ventricular ejection fraction; LVESVi, left ventricular end-systolic volume indexed; LV ICMi, left ventricular intracellular mass indexed; LVMi, left ventricular mass indexed.

*P < 0.05.

**Figure 2:**
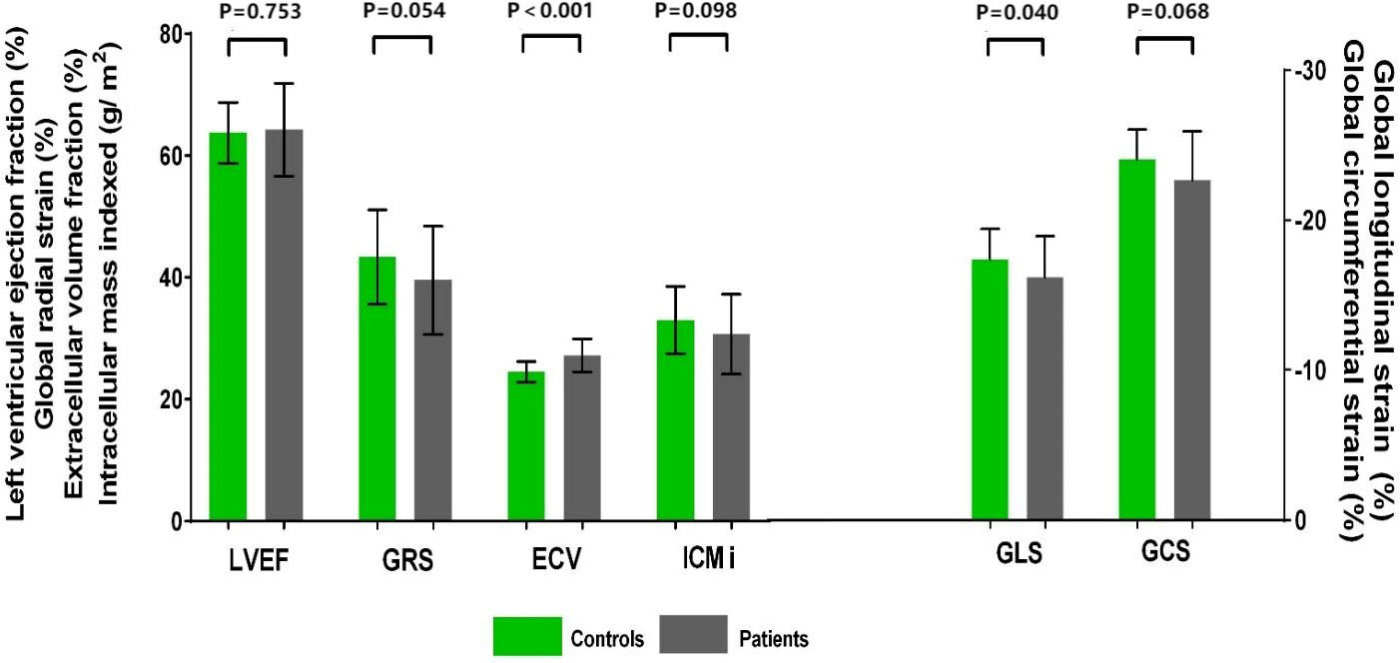
Cardiovascular magnetic resonance data in patients with gynecologic cancer and healthy control subjects. LVEF did not differ between patients with gynecologic cancer and healthy control subjects. Patients had higher GLS and ECV. A trend toward higher GCS and lower GRS and ICMi was observed in patients. ECV, extracellular volume fraction; GCS, global circumferential strain; GLS, global longitudinal strain; GRS, global radial strain; LVEF, left ventricular ejection fraction.

Patients had a higher LV GLS compared with control subjects (−16.2% ± 2.8% vs. −17.4% ± 2.0%, *P* = 0.040); a trend toward higher LV GCS (median −23.4% [IQR −24.8% to −21.3%] vs. median −24.3% [IQR −25.6% to −21.8%], *P* = 0.068) and lower LV GRS (39.5% ± 8.9% vs. 43.3% ± 7.7%, *P* = 0.054) was observed in patients (**Figure 2**). Patients with two or more chemotherapy regimens had significantly higher GCS compared with both patients with one regimen (−21.4% ± 4.0% vs. −23.7% ± 2.1%, *P* = 0.004) and the control group (−21.4% ± 4.0% vs. −24.0% ± 2.0%, *P* = 0.003). Patients treated with anthracycline-containing chemotherapy and non-anthracycline chemotherapy had similar GLS (−15.9% ± 2.1% vs. −16.2% ± 2.9%, *P* = 0.744).

### 3.3 Markers of myocardial fibrosis

**Figure 3:**
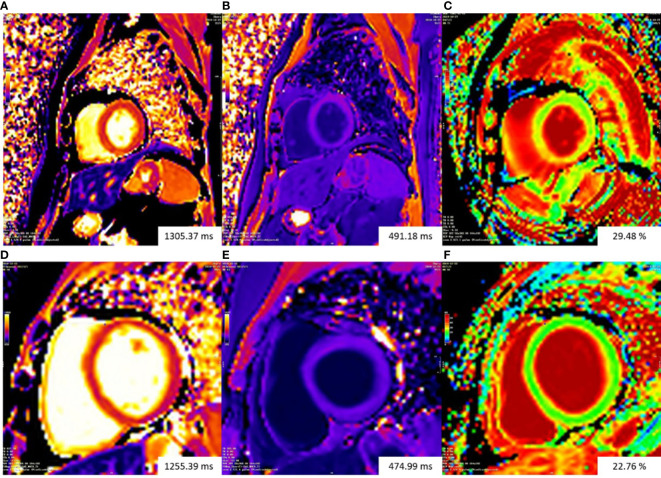
Representative examples of T1 mapping. The color bars of all T1 maps are exhibited on the upper left quarter of figures. Changes in color from the bottom to the top of the color bar correspond to value increases. Top row: patient with gynecologic cancer undergoing chemotherapy had increasing ECV (29.48%) **(C)**. Bottom row: ECV mapping were homogeneous in a normal control subject (22.76%) **(F)**.The Native T1 value of the patient **(A)** is slightly higher than that of the normal control subject **(D)**. The patient **(B)** and the normal control subject **(E)** had similar post-contrast T1 value. ECV, extracellular volume fraction.

A total of 58 patients with gynecologic cancer and 30 healthy control subjects consented to receive gadolinium injection for ECV estimation. Native T1 did not differ between patients and control subjects (median 1273 ms [IQR 1251–1298 ms] vs. median 1267 ms [(IQR 1243–1283 ms), *P* = 0.176]. The ECV was higher in patients than in control subjects (27.2% ± 2.7% vs. 24.5% ± 1.7%; *P* < 0.001) (**Figures 2**, **3**). A total of 21 (36.2%) patients had an ECV ≥ 2 SDs above the mean ECV value for controls. ECV was increased in both patients treated with non-anthracycline chemotherapy (27.2% ± 2.8% vs. 24.5% ± 1.7%; *P* < 0.001) and patients treated with anthracycline-containing chemotherapy (26.9% ± 2.4% vs. 24.5% ± 1.7%; *P* = 0.001) compared with the control group. A trend toward lower ICMi (median 30.46 g/m^2^ [IQR 25.50–36.40 g/m^2^] vs. median 32.79 g/m^2^ [IQR 27.74–35.76 g/m^2^]; *P* = 0.098) was observed in patients. HighC group had significantly lower ICMi compared with both lowC group (median 27.44 g/m^2^ [IQR 24.03–31.15 g/m^2^] vs. median 34.30 g/m^2^ [IQR 29.93–39.79 g/m^2^]; *P* = 0.002) and the control group (median 27.44 g/m^2^ [IQR 24.03–31.15 g/m^2^) vs. median 32.79 g/m^2^ [27.74–35.76 g/m^2^]; *P* = 0.002). Patients with two or more chemotherapy regimens had significantly lower ICMi compared with both patients with one chemotherapy regimen (27.45 ± 5.16 g/m^2^ vs. 33.32 ± 6.42 g/m^2^; *P* < 0.001) and the control group (27.45 ± 5.16 g/m^2^ vs. 33.02 ± 5.52 g/m^2^; *P* < 0.001).

Native T1, ECV and ICMi did not correlate with LVEF. The ECV was found to be inversely correlated with LVMi (*r* = −0.262, *P* = 0.047). ICMi was positively correlated with LV mass to volume ratio (*r* = 0.497, *P* < 0.001). No parameter of global strain correlated with native T1, ECV, or ICMi.

**Figure 4:**
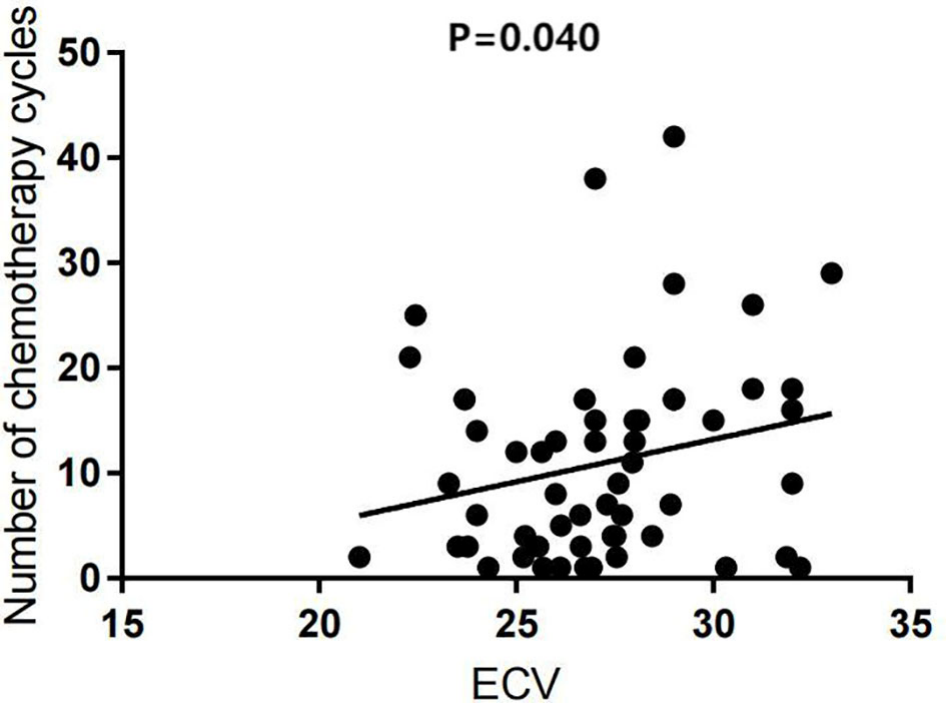
Scatter diagram of extracellular volume fraction and number of chemotherapy cycles. ECV, extracellular volume fraction.

**Figure 5:**
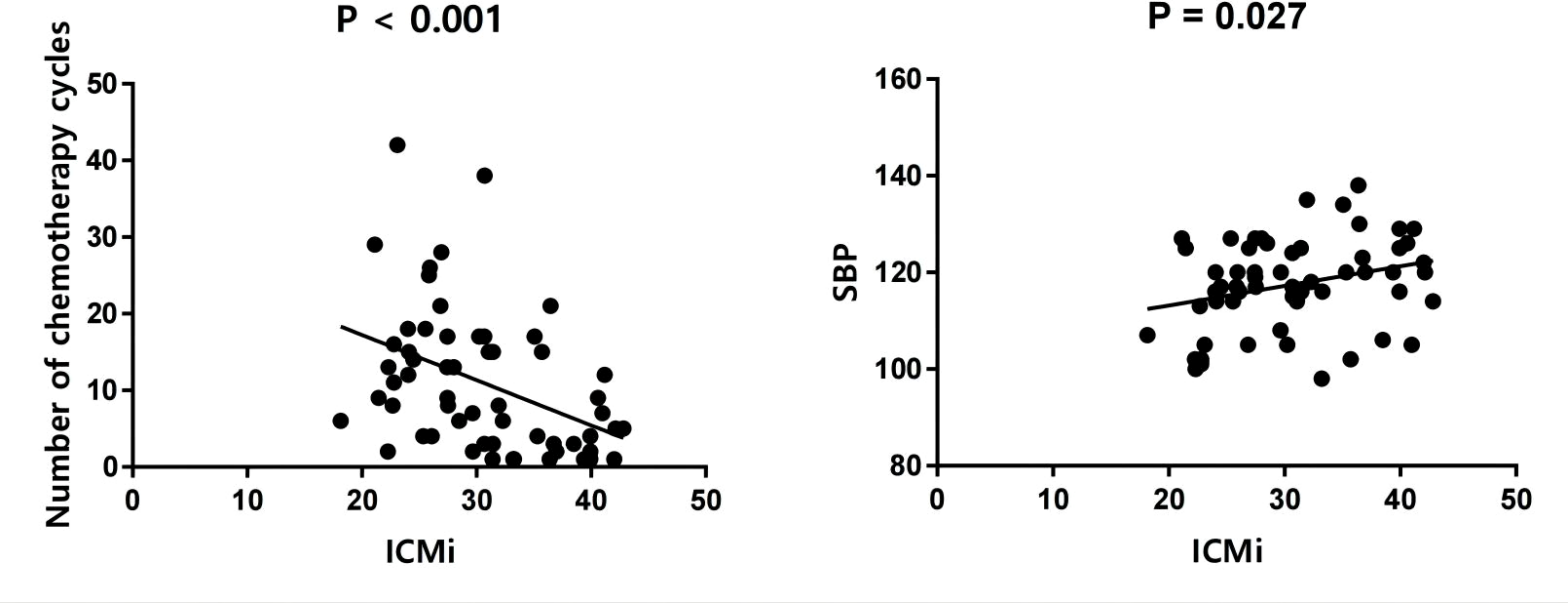
Scatter diagram of intracellular mass indexed and number of chemotherapy cycles and systolic blood pressure. ICMi, intracellular mass indexed; SBP, systolic blood pressure.

Univariate analysis showed that the ECV was positively correlated with the number of chemotherapy cycles (*r* = 0.270, *P* = 0.040) (**Figure 4**). In the multivariate analysis, which was adjusted for chemotherapy drugs and clinical confounders, the number of chemotherapy cycles was independently associated with an increase in the ECV (Standard regression coefficient [β] = 0.383, *P* = 0.014) (**Table 3**). Univariate analysis showed that the ICMi was positively correlated with systolic blood pressure (*r* = 0.290, *P* = 0.027) and inversely correlated with number of chemotherapy cycles (*r* = −0.461, *P* < 0.001) (**Figure 5**), bevacizumab (*r* = −0.342, *P* = 0.009) and age (*r* = −0.275, *P* = 0.037). In the multivariate analysis, the number of chemotherapy cycles was associated with a decrease in the ICMi (β = -0.349, *P* = 0.009) (**Table 4**). Sensitivity analysis showed that anthracycline was not associated with either ECV or ICMi (**Supplementary Tables 1**, **2**).

**Table 3:** The association between chemotherapy and left ventricular extracellular volume fraction.

	Univariable Analysis		Multivariable Analysis	
	r	P value	β	P value
Number of chemotherapy cycles	0.270	0.040 *	0.383	0.014 *
Anthracycline	0.027	0.839	-0.115	0.464
Bevacizumab	0.082	0.543	-0.055	0.737
Cyclophosphamide/ifosfamide	0.117	0.381	-0.012	0.939
Age	0.104	0.437	0.042	0.767
Body mass index	-0.041	0.758	-0.031	0.836
Heart rate	0.132	0.335	0.238	0.133
Systolic blood pressure	-0.125	0.350	-0.244	0.114

*P < 0.05.

**Table 4:** The association between chemotherapy and left ventricular intracellular mass indexed.

	Univariable Analysis		Multivariable Analysis	
	r	P value	β	P value
Number of chemotherapy cycles	-0.461	<0.001 *	-0.349	0.009 *
Anthracycline	-0.046	0.730	0.003	0.980
Bevacizumab	-0.342	0.009 *	-0.244	0.086
Cyclophosphamide/ifosfamide	0.014	0.919	0.279	0.049 *
Age	-0.275	0.037 *	-0.233	0.061
Body mass index	-0.093	0.486	-0.036	0.781
Heart rate	-0.128	0.350	-0.144	0.283
Systolic blood pressure	0.290	0.027 *	0.341	0.011 *

*P < 0.05.

Among 58 patients with gynecologic cancer and 30 healthy control subjects consented to receive gadolinium injection, 16 patients (16/58, 27.6%) were recorded as positive for LGE, as opposed to healthy control subjects who were all negative. Patients who were positive for LGE had higher ECV than healthy control subjects (27.72 ± 2.47% vs. 24.49 ± 1.70%; *P* < 0.001). Patients who were negative for LGE also had higher ECV (26.96 ± 2.78% vs. 24.49 ± 1.70%; *P* < 0.001) than healthy control subjects.

## 4 Discussion

The main findings of this study were as follows: 1) In comparison with their healthy peers, ECV were elevated in patients with gynecologic cancer and low cardiovascular risk undergoing chemotherapy, and in more than one-third of patients showing an expansion of ECV. 2) The number of chemotherapy cycles was associated with an increase in the ECV, indicating that ECV expansion was obvious with the increase of chemotherapy load. 3) The number of chemotherapy cycles was associated with a decrease in the ICMi; and in patients with higher chemotherapy cycles (> 6 cycles) and patients with more than one chemotherapy regimen, the decrease in ICMi was more prominent. It is possible that myocyte loss is part of the mechanism of ECV expansion in patients with higher chemotherapy load, although this needs further confirmation. 4) On average, patients with gynecologic cancer and low cardiovascular risk undergoing chemotherapy have preserved systolic function, although GLS is higher in these patients compared with healthy control subjects. Patients with more than one chemotherapy regimen had higher GCS.

Antineoplastic therapy is frequently complicated by the development of cardiotoxicity (3), which may have the potential to offset the gains in survival obtained with these cancer treatment advances (15, 16).

Novel tissue characterization sequences, such as native T1 mapping and ECV estimation, have shown promise for the detection of diffuse fibrosis and inflammation/edema by quantitatively assessing its presence and extent (6). Previous studies have demonstrated that increased ECV could be identified with CMR in cancer survivors and those currently undergoing treatment (9, 12, 13, 17, 18). Our study found that ECV was elevated in cancer patients undergoing chemotherapy, which was consistent with the results of prior reports. In previous ECV studies, the cancer types of the subjects were mainly breast cancer, lymphoma, hematologic malignancy, sarcoma, bone cancer, and childhood cancer (9, 12, 13, 17–22). Different from previous studies, the subjects of our study were patients with gynecologic cancer. To the best of our knowledge, ours is the first study to characterize Native T1 and ECV as candidate markers of myocardial fibrosis in patients with gynecologic cancer treated with chemotherapy.

In chronic processes, an increase in ECV can reflect an expansion of the extracellular space, as observed in reactive fibrosis, or it can be due to cellular atrophy and myocyte death, which results in replacement fibrosis (17). Our study found that number of chemotherapy cycles was associated with an increase in the ECV and a decrease in the ICMi. Moreover, in patients with higher chemotherapy cycles and patients with more than one chemotherapy regimen, the decrease in ICMi was more prominent. Our finding indicated that the ECV expansion was obvious with the increase of chemotherapy load and myocyte loss might be the prevailing mechanism of ECV expansion in these patients, which was similar to findings of some previous studies. In a recent study by Mawad et al. (17), ICMi was lower in childhood cancer survivors compared with controls. In a study conducted by Harries et al. (18), anthracycline-treated cancer survivors with normal LVEF have significant perturbations of myocardial cell volume compared with controls.

In previous studies on imaging evaluation of cardiotoxicity of taxane plus platinum-based chemotherapy, echocardiography was used for imaging evaluation (23, 24). These studies concluded that paclitaxel and carboplatin combination could induce subtle impairment in myocardial mechanical function which can be detected by advanced deformation imaging techniques, and paclitaxel has cardiotoxic effects. The literature on CMR evaluation in these population is lacking. Most previous studies of CMR T1 mapping in chemotherapy patients focused on patients who received anthracycline-based chemotherapy, and there have been few studies on non-anthracycline chemotherapy. Unlike previous studies, our research subjects primarily consisted of patients treated with non-anthracycline chemotherapy, among which the taxane plus platinum regimen was the most commonly used. Our results indicated that ECV was increased in both nonanthracycline-treated patients and anthracycline-treated patients compared with the control group. This finding is inconsistent with the results of previous studies. In a study by Jordan et al. (12), the ECV of nonanthracycline-treated survivors (29.5% ± 1.0%) was no different from that of pretreatment survivors (27.8% ± 0.7%) and controls (26.9% ± 0.2%). In addition, in a study by Meléndez et al. (13), ECV was not significantly elevated at 3 months after treatment initiation compared with baseline in subjects receiving non-anthracycline-based chemotherapy. Therefore, we believe that a larger study is necessary to determine the relationship between non-anthracycline chemotherapy and changes in myocardial fibrosis.

The ECV value of the chemotherapy patients in our study was 27.2% ± 2.7%, which was lower than that reported in the research conducted by Jordan et al. (12). This is likely because our study excluded individuals with cardiovascular risk factors, such as coronary heart disease, diabetes, and uncontrolled hypertension. In comparison with our study, previous research conducted by Jordan et al. included cancer survivors treated with chemotherapy and cancer-free control participants with and without risk factors for cardiovascular fibrosis and demonstrated a higher ECV value in anthracycline-treated cancer participants (30.4% ± 0.7%) compared with cancer-free comparators (26.9% ± 0.2%).

On average, systolic function was relatively preserved in patients with gynecologic cancer in our study. We found that compared with healthy control subjects, GLS was impaired in patients. In addition, we observed a trend toward impaired GCS and GRS in the patients, whereas LVEF did not differ between the controls and patients. Systematic review and meta-analysis confirmed the value of echocardiography-derived GLS for the early detection of myocardial changes and prediction of cardiotoxicity in patients treated with chemotherapy (25, 26). In a recent study by Houbois (27), changes in the CMR strain were prognostic for subsequent cancer therapy-related cardiac dysfunction. Although the predictive value of our findings is presently unclear, it is possible that the CMR-derived strain has the potential to play a significant role in the early detection of cardiotoxicity in patients with gynecologic cancer.

This study has several limitations that need to be addressed. First, this was a single-center study with a relatively low number of patients. The cross-sectional design of the study precludes the assessment of temporal changes in patients with gynecologic cancer treated with chemotherapy. Moreover, our study did not include cancer patients who were chemotherapy-naïve. Because the presence of cancer is independently associated with alterations in cardiac native T1 (28), we could not exclude the possible role of tumor-induced cardiac fibrosis in these patients. Therefore, future studies with a longitudinal design comparing CMR parameters in each case before and after treatment are warranted. Second, not all the inpatients were invited to participate, and a selection bias cannot be excluded. Third, the lack of tissue specimens prevented a correlation between ECV and the histological gold standard for myocardial fibrosis, although previous studies have demonstrated this association (29, 30). Expansion of the myocardial interstitial space could occur in the presence of inflammation and edema, or as interstitial fibrosis initiates within the extracellular matrix, or in the presence of myocardial tissue volume reduction (due to cardiomyocyte atrophy or loss). In our study, the median interval from the last chemotherapy cycle and the CMR execution was 19.0 days (IQR 10.3-25.8), which could not rule out that the elevation of ECV was partly due to myocardial edema. Therefore, future studies involving myocardial biopsies would be helpful. Fourth, the long-time follow-up outcomes have not yet been established in these cancer patients. Future studies are required to determine whether these patients with elevated ECV experience higher rates of cardiovascular events. Furthermore, we didn’t include patients receiving immunotherapy. Immune checkpoint inhibitors (ICIs) have led recent advances in the field of cancer immunotherapy improving overall survival in multiple malignancies. Future studies are required to explore the cardiotoxicity of chemotherapy and immunotherapy in patients with gynecologic cancer (31).

In conclusion, patients with gynecologic cancer and low cardiovascular risk who undergo chemotherapy have diffuse extracellular volume expansion, which is obvious with the increase of chemotherapy cycles. Myocyte loss may be part of the mechanism in patients with a higher chemotherapy load.

## Data availability statement

The raw data supporting the conclusions of this article will be made available by the authors, without undue reservation.

## Ethics statement

The studies involving human participants were reviewed and approved by Research Ethics Board of West China Second University Hospital. The patients/participants provided their written informed consent to participate in this study.

## Author contributions

Y-KG, R-TY, HL have substantial contributions to the conception or design of the work; LYe, D-QW, M-XY, Q-LL, X-JL, K-ML, LS, YM, H-QH, LZ, LYa, J-JZ, F-MG, H-YX, L-JX have substantial contributions to the acquisition, analysis, or interpretation of data for the work. LYe, D-QW, M-XY, Q-LL, X-JL, K-ML, H-YX, L-JX have contributions of drafting the work; LS, YM, H-QH, LZ, LYa, J-JZ, F-MG, Y-KG, R-TY, HL have contributions of revising it critically for important intellectual content. All these authors have final approval of the version to be published and agreement to be accountable for all aspects of the work in ensuring that questions related to the accuracy or integrity of any part of the work are appropriately investigated and resolved.
