# Supplementary material for: Chemotherapy effect on myocardial fibrosis markers in patients with gynecologic cancer and low cardiovascular risk
Source: Front Oncol. 2023 Aug 8;13:1173838. doi: 10.3389/fonc.2023.1173838 (PMC10442931; doi:10.3389/fonc.2023.1173838)
Supplement: Supplementary file 1 [file Table_1.pdf]

Supplementary table 1
The association between chemotherapy agents and left ventricular extracellular volume fraction

	Multivariable Analysis	
	**β**	***P* value**
Anthracycline	-0.114	0.479
Bevacizumab	-0.055	0.705
Cyclophosphamide/ ifosfamide	0.094	0.561

Supplementary table 2
The association between chemotherapy agents and left ventricular intracellular mass indexed

	Multivariable Analysis	
	**β**	***P* value**
Anthracycline	0.009	0.954
Bevacizumab	-0.394	0.005*
Cyclophosphamide/ ifosfamide	0.138	0.360

* P < 0.05.
